# Complete Genome Sequence of *Mycobacterium chelonae* Type Strain CCUG 47445, a Rapidly Growing Species of Nontuberculous Mycobacteria

**DOI:** 10.1128/genomeA.00550-16

**Published:** 2016-06-09

**Authors:** Daniel Jaén-Luchoro, Francisco Salvà-Serra, Francisco Aliaga-Lozano, Carolina Seguí, Antonio Busquets, Antonio Ramírez, Mikel Ruíz, Margarita Gomila, Jorge Lalucat, Antoni Bennasar-Figueras

**Affiliations:** a Microbiology, Biology Department, Universitat de les Illes Balears, Palma de Mallorca, Spain; b Department of Infectious Diseases, Sahlgrenska Academy, University of Gothenburg, Gothenburg, Sweden; c Clinica Rotger, Microbiologia, Palma de Mallorca, Spain; d Hospital Universitario Son Espases (HUSE), Palma de Mallorca, Spain; e Microbiologia, Institut Mediterrani d’Estudis Avançats (IMEDEA, CSIC-UIB), Esporles, Spain; f Microbiologia, Instituto Universitario de Investigaciones en Ciencias de la Salud (IUNICS-UIB), Universitat de les Illes Balears, Palma de Mallorca, Spain

## Abstract

*Mycobacterium chelonae* strains are ubiquitous rapidly growing mycobacteria associated with skin and soft tissue infections, cellulitis, abscesses, osteomyelitis, catheter infections, disseminated diseases, and postsurgical infections after implants with prostheses, transplants, and even hemodialysis procedures. Here, we report the complete genome sequence of *M. chelonae* type strain CCUG 47445.

## GENOME ANNOUNCEMENT

*Mycobacterium chelonae*, taxonomically elevated as a species in 1992 (1), is a nonpigmented rapidly growing *Mycobacterium* sp., which is widely distributed in the environment and can be isolated from soils and waters worldwide (2). Its high resistance to commonly used antibiotics (3) and disinfectants (4) allows *M. chelonae* to colonize urban and hospital water systems, even from strictly controlled water used for hemodialysis (5). This fact explains its relatively easy access to susceptible humans. *M. chelonae* is a recognized opportunistic pathogen commonly related to skin infections and has been responsible for tattoo infection outbreaks (6, 7), as well as localized cellulitis, abscesses, osteomyelitis, catheter infections, and disseminated diseases (8). This microorganism is also involved in surgical infections of implants with prostheses or devices, transplants, and hemodialysis processes (9–11). Although pulmonary infections by *M. chelonae* are not common, there are reported cases of them in patients with an underlying pulmonary disease, for instance, cystic fibrosis (12). The elderly and those with immunocompromised states have the worst prognoses when an infection appears and mycobacteria are involved (13). The aim of this project is to present the complete genome sequence of *Mycobacterium chelonae* type strain CCUG 47445, a highly drug-resistant member of the nontuberculous mycobacteria group.

DNA was extracted from a 4-day culture grown at 30°C on R2A using the Wizard genomic DNA purification kit (Promega, Fitchburg, WI, USA) after cell disruption using a FastPrep-24 (MP Biomedicals, CA). Whole-genome sequencing was done using an Illumina HiSeq 2500 platform (2 × 100 cycles, paired-end, insert size of 250 to 350 ± 50 bp), obtaining 7,526,798 reads, and a Pacific Biosciences (PacBio RSII) platform using 10-kb SMRTbell libraries, resulting in 136,465 reads with an average length of 2.266 bp.

The Illumina reads were trimmed and assembled with CLC Genomics Workbench version 6.5.1 (CLC bio, Aarhus, Denmark). The draft assembly yielded 85 contigs. The optimal k-mer size was automatically determined using KmerGenie (14). These contigs were used to perform a hybrid scaffolding process based on alignment of the PacBio contiguous long reads (CLRs). This alignment and the determination of order, orientation, and distance between contigs was done using the program SSPACE-LongRead version 1.0 (15). Gaps were closed using GapFiller version 1.10 (16), based on high-quality Illumina paired-end reads.

The result was a completely closed genome of 5,029,817 bp, with a 63.92% G+C content. According to the NCBI Prokaryotic Genome Annotation Pipeline (PGAP) (17), this genome contains 4,726 predicted coding sequences (CDSs). A unique complete rRNA operon and 48 tRNAs were found. The genome was also analyzed using the Comprehensive Antibiotic Resistance Database (CARD) (18) and RAST annotation server (19). The annotation methods highlight 26 putative resistance genes against antibiotics (i.e., beta-lactams and rifampicin), as well as several multidrug transporters, 21 genes for heavy metals resistance, 74 potential genes related to virulence operons, 2 putative genes related to pathogenic islands, and 2 genes related to persistent cells capability.

The genome sequence of *M. chelonae* CCUG 47445^T^ represents essential data for phylogenomic studies and will be useful for understanding its pathogenic capability and resistance profile to environmental stress factors.

### Nucleotide sequence accession number.

This complete genome sequence has been deposited at DDBJ/ENA/GenBank under the accession no. CP007220.
